# Mapping Dementia Care in U.S. Prisons: A State-Level Characterization of Policies and Programs

**DOI:** 10.1093/geroni/igaf122.4158

**Published:** 2025-12-31

**Authors:** Elizabeth Breen, Indrakshi Roy, Ricky Camplain

**Affiliations:** Indiana University School of Medicine, Indianapolis, Indiana, United States; Indiana University, Bloomington, Bloomington, Indiana, United States; Indiana University Bloomington, Bloomington, Indiana, United States

## Abstract

The aging of the U.S. prison population has led to a marked increase in incarcerated individuals with Alzheimer’s disease and related dementias (ADRD). Despite the clinical complexity and behavioral management needs associated with dementia, carceral health systems remain underprepared to deliver appropriate care. Research on ADRD in correctional settings is sparse, and national policy guidelines are lacking. Because healthcare in prisons is administered at the state level, understanding the landscape of dementia care requires granular, jurisdiction-specific analysis. This project presents a comprehensive, state-by-state review of dementia-related services in U.S. carceral systems. Public records, correctional health documents, and secondary sources were used to categorize each state based on (1) presence of a dementia-specific program, (2) availability of geriatric or hospice care units, and (3) use of compassionate or geriatric release policies. Our analysis reveals wide variability: only 34% of states currently operate dementia-specific prison programs, while others rely on general end-of-life services or ambiguous early release frameworks. Many states have no identifiable infrastructure for supporting cognitive decline among incarcerated individuals. These findings highlight critical gaps in both the delivery and documentation of dementia care in prisons. As the incarcerated population continues to age, developing scalable, evidence-informed strategies for the management of ADRD in carceral settings will be essential for ensuring continuity of care, safety, and resource stewardship across the health and justice systems.

